# Supplementary material for: Tissue libraries enable rapid determination of conditions that preserve antibody labeling in cleared mouse and human tissue
Source: eLife. 2023 Jan 19;12:e84112. doi: 10.7554/eLife.84112 (PMC9889093; doi:10.7554/eLife.84112)
Supplement: Supplementary file 1. [file elife-84112-supp1.docx]

| Sample Number | Sex | Age at Death | Braak Staging | Hemisphere | Comorbidities | Postmortem Interval Before Fixation |
| --- | --- | --- | --- | --- | --- | --- |
| 1 (1581) | M | 89 | VI | Frozen Left | CVD and CAA | ~12 years |
| 2 (1762) | F | 71 | VI | Frozen Right | CVD and CAA | ~9 years |
| 3 (2620) | M | 52 | N/A | Right | N/A | <24 hours |

**Supplementary table 1.** Demographic and characteristics of all subjects used in this study including sex (M = Male ; F = Female), Age at death, Braak stage, Hemisphere, CerebroVascular Disease (CVD), and Cerebral Amyloid Angiopathy (CAA).
